# Modification of Covalent Triazine-Based Frameworks for Photocatalytic Hydrogen Generation

**DOI:** 10.3390/polym14071363

**Published:** 2022-03-27

**Authors:** Jijia Xie, Zhiping Fang, Hui Wang

**Affiliations:** 1 Sinopec Beijing Research Institute of Chemical Industry, Beijing 100029, China; 2 Department of Science & Technology R & D, Sinopec Group, Beijing 100728, China; fangzp@sinopec.com; 3 Department of Chemical Engineering, University College London, London WC1E 7JE, UK

**Keywords:** covalent triazine-based frameworks, polymeric photocatalyst, photocatalysis, hydrogen generation

## Abstract

The conversion of solar energy and water to hydrogen via semiconductor photocatalysts is one of the efficient strategies to mitigate the energy and environmental crisis. Conjugated polymeric photocatalysts have advantages over their inorganic counterparts. Their molecular structures, band structures, and electronic properties are easily tunable through molecular engineering to extend their spectral response ranges, improve their quantum efficiencies, and enhance their hydrogen evolution rates. In particular, covalent triazine-based frameworks (CTFs) present a strong potential for solar-driven hydrogen generation due to their large continuous π-conjugated structure, high thermal and chemical stability, and efficient charge transfer and separation capability. Herein, synthesis strategies, functional optimization, and applications in the photocatalytic hydrogen evolution of CTFs since the first investigation are reviewed. Finally, the challenges of hydrogen generation for CTFs are summarized, and the direction of material modifications is proposed.

## 1. Introduction

The ability to effectively harness and store renewable energy in chemical form has been widely recognized as a promising and sustainable strategy to meet future worldwide energy demands. Solar energy is by far one of the most widely distributed renewable primary energy sources. As a secondary energy source, hydrogen energy is generally considered an ideal energy carrier due to its high energy density, zero emissions, storability, and transportability. Therefore, converting solar energy into hydrogen in a single step is of great significance for the efficient and clean utilization of solar energy, reducing carbon emissions and environmental pollution [1,2].

As photocatalysts, semiconductors can effectively convert solar energy into chemical energy by adsorping photons to generate photoelectrons and then reducing protons in water to hydrogen [3,4]. Over the past 40 years, inorganic semiconductors such as metal oxides and metal sulfates have been widely investigated. However, limited by their relatively fixed crystal structure, the band structures of such inorganic materials are difficult to adjust to enhance the range of light adsorption and the capability of charge transportation [5,6]. In contrast, a conjugated structure allows polymers to be easily regulated by molecular engineering methods such as rearranging polymeric scaffolds and functionalizing them with substituents to adjust their band structure, enhance their charge transfer, and improve their dispersibility in water. Therefore, the photocatalytic activity of polymeric semiconductors is able to promote solar hydrogen generation more than inorganic catalysts [7].

**Figure 1:**
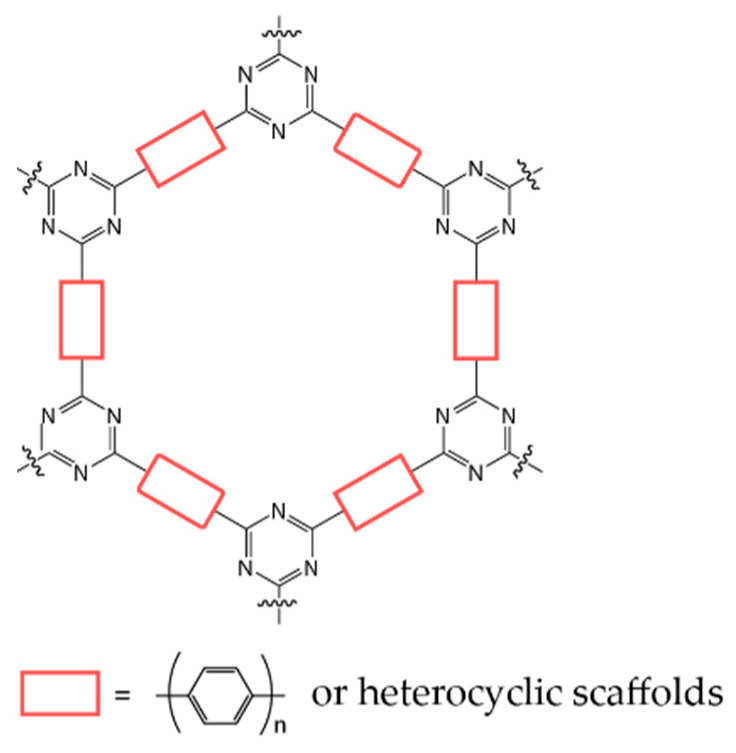
Sketch of the structure of covalent triazine-based frameworks.

The first investigation of covalent triazine-based frameworks for photocatalytic hydrogen production attracted many researchers due to their structural tunability and inherent porosity [8]. Since then, the discovered high electron mobility of triazine-based frameworks allowed them to be a great block [9]. As shown in Figure 1, triazine-based frameworks (CTFs) are polymeric semiconductors composed of alternating triazine and benzene motifs. Compared with other widely researched polymeric photocatalysts such as graphitic carbon nitride (g-C_3_N_4_), conjugated heterocyclic polyamine, etc., CTFs (1) have a large π-conjugated structure, resulting in high chemical stability and high flexibility for the transportation of photocharges in the delocalized π bond [10,11]; (2) contain lone-pair electrons in the nitrogen sites of triazine rings, which can be easily excited to form an n⟶π transition and high-density photoelectrons [12,13]; and (3) by changing the monomer, are flexibly modified to increase the length of polymer backbones and functionalize them with substitutes, thus optimizing the band structure [14], as well as enhancing hydrophilicity [15] and the ability of hydrogen desorption [16].

Therefore, CTFs have become one of the key materials in photocatalytic hydrogen generation and have recently attracted worldwide attention due to their unique electronic structure, excellent chemical stability, and tunable molecular design properties. However, studies on structure–activity relationships, reaction mechanisms, and photogenerated charge kinetics are still at a relative infant stage. By designing new monomers and optimizing polymerization conditions, a series of CTFs with different physical and chemical structures have been synthesized. Combined with photoelectrochemical spectra and first-principle calculations, the relationship between photocatalyst structure and hydrogen evolution performance has been estimated. Polymerization conditions, functionalization, and the doping of heteroatoms may also impact the composition of defects in backbones and end groups, the degree of polymerization, crystallinity, conjugation, carbonization, and the capability of hydrophilicity, all of which play significant roles in photocatalytic activity. However, the synthesis of CTFs requires harsh conditions, including high temperature and acid purification, easily resulting in structural disorder or destruction. Hence, it remains challenging to develop efficient CTFs with stable structures. Therefore, in this manuscript, the effects of different preparation methods and polymerization paths of CTFs in the related literature since the first investigation in 2014 until now are summarized. Then, strategies to control band structure and enhance charge transfer/separation are reviewed. The challenges of improving hydrophilicity, increasing activation energy and prolonging the lifetime of photocharges are discussed. The potential to drive overall solar water splitting by designing a new structure of CTFs, decorating cocatalysts, and constructing heterojunctions is also proposed.

## 2. Synthesis of CTFs

**Figure 2:**
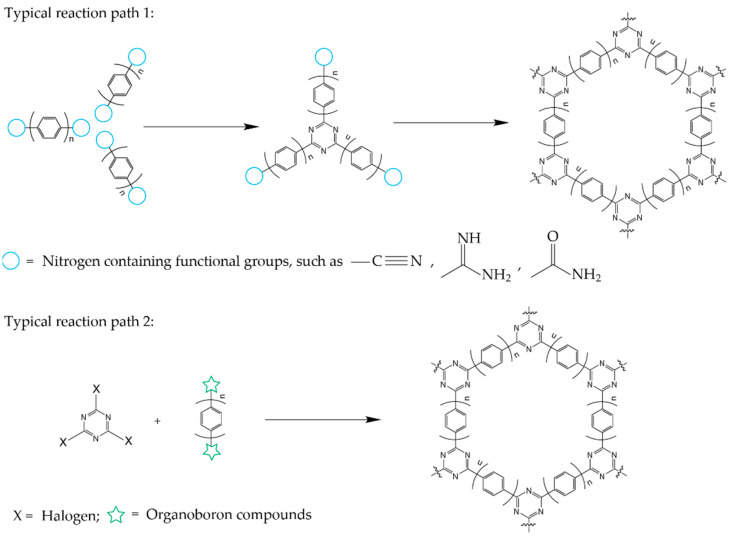
Typical reaction paths for the synthesis of CTFs.

There are two main polymerization paths to synthesize CTFs, as shown in Figure 2. One is to synthesize triazine rings by cyano addition trimerization or amino dehydration trimerization. The other is to form the basic backbone via Suzuki coupling, Friedel–Crafts alkylation, and other reactions by triazine-containing monomers. Major CTFs have been synthesized by four reaction methodologies, including ionothermal synthesis [17,18,19,20], microwave-assisted synthesis [10,21,22], dehydration polycondensation [23,24], and solution polymerization [22,25,26,27,28,29]. Here, various strategies of CTF synthesis are discussed, including ionothermal synthesis, microwave, dehydration polycondensation, and solution polymerization.

### 2.1. Ionothermal Synthesis

**Figure 3:**
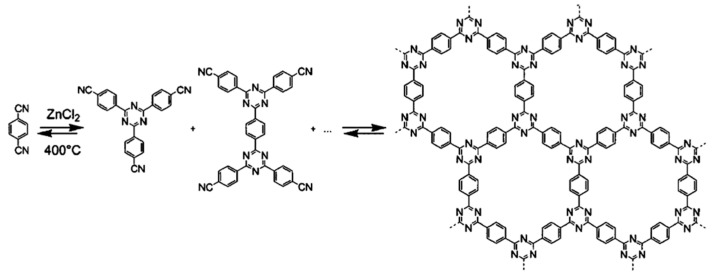
Polymerization of CTF-1 from the monomer 1,4-dicyanobenzene via ionothermal synthesis [17].

Ionothermal synthesis uses hot ionic liquids as solvent and catalyst for the polymerization of monomer precursors, which are mainly utilized for the trimerization of aromatic nitriles, such as 1,3,5-benzenetrinitrile, terephthalonitrile, 2,6-dicyanonaphthalene, etc. High polymerization temperatures over 400 °C and anaerobic environments are typically required to drive such free radical reactions. This synthesis method was first proposed by Kuhn et al. in 2008 [17], as the first physical synthesis of CTFs. However, as C≡N triple bonds are thermodynamically much more stable than C-H bonds in the aromatic and heterocyclic backbones, the backbones were easily decomposed during polymerization, resulting in the collapse of the framework [30]. Meanwhile, the trimerization of cyano groups is an exothermic reaction. The reaction equilibrium constant decreased with the increase in temperature, proceeded toward the reverse, and the degree of polymerization decreased. Therefore, it was necessary to use a proper catalyst to reduce the activation temperature of cyano groups during polymerization and ensure the integrity of the framework. Since N atoms in cyano groups contain lone-pair electrons, which are a type of Lewis base, Lewis acid molten salts were utilized as catalyst and solvent to reduce the activation energy of the cyano group via acid–base reaction [17]. Except for ZnCl_2_, the melting temperature of most Lewis acid molten salts is concentrated at 600–700 °C, while the C-H bonds in the monomers are prone to cracking over 400 °C [30]. Therefore, ZnCl_2_ molten salt is typically selected as a solvent and catalyst to drive polymerization. For a typical synthesis, as shown in Figure 3, the monomer terephthalonitrile and the catalyst ZnCl_2_ were first sealed in a vacuum quartz ampoule at a ratio of 1:10. Then, the sealed ampoule was placed in a tubular furnace at 400 °C for 40 h to obtain a fully polymerized framework, referred to as CTF-1 [17]. During the reaction, -C≡N triple bonds were activated by Lewis acid, and one of the electrons was attracted to form ·C=N radicals. Then, a triazine was able to be formed via the trimerization of three ·C=N radicals. As only cyano groups participate in the reaction, various monomers with two or more cyano groups could be utilized to form the triazine-based frameworks.

By changing the monomer, ZnCl_2_ molten salt has also proved to be able to drive the polymerization of more than ten kinds of triazine-based polymers containing aromatic rings or heterocycles such as pyridine, bipyridine, and thiophene. However, among all synthesized CTFs, only CTF-1 can maintain high crystallinity and permanent mesopores and micropores. Recently, a new monomer 2,6-dicyanonaphthalene was synthesized by cyaniding naphthalene via Ullmann coupling in order to obtain a highly crystallized CTF with larger pore size, referred to as CTF-2 [19].

Briefly, ionothermal synthesis has been widely utilized for a variety of monomers for different types of CTF synthesis, which facilitates the modification of materials in subsequent applications [18]. Such polymers generally had a high surface area of approximately 1000 m^2^ g^−1^ [18,19]. In addition, some polymerized materials such as CTF-1 and CTF-2 were highly crystallized with permanently fixed-sized mesopores and micropores, which benefited from subsequent catalytic reactions. However, ionothermal synthesis requires a high temperature and long reaction time; thus, carbonization and coking are difficult to avoid during polymerization [20]. The generated amorphous carbon resulting from the high reaction temperature can block the light absorption of the catalyst during photocatalytic reactions. Meanwhile, ionothermal reactions must be conducted under a vacuum atmosphere and require a long reaction time, which is not conducive to the batch preparation of catalysts and limits the scale-up of subsequent applications.

### 2.2. Microwave-Assisted Synthesis

**Figure 4:**
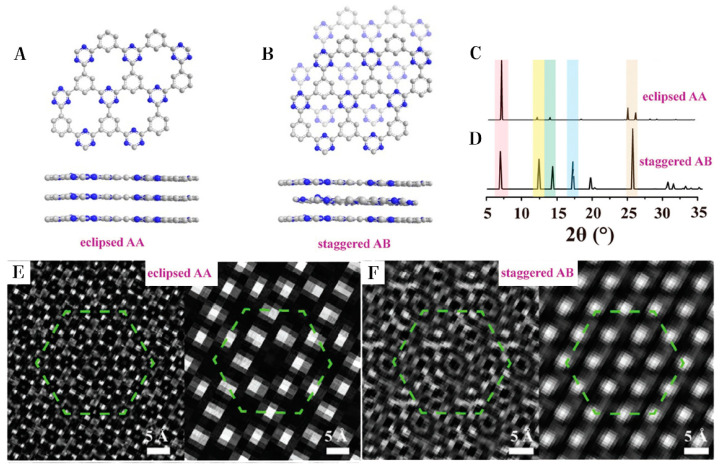
Two possible stacking arrangements with their (**A**,**B**) estimated structure, (**C**,**D**) calculated XRD, and (**E**,**F**) simulated HRTEM [31]. Note: (**A**,**C**,**E**) eclipsed AA stacking; (**B**,**D**,**F**) staggered AB stacking.

Inspired by ionothermal synthesis, microwave-assisted synthesis was developed. Microwave synthesis can enhance the hot-spot temperature and improve the polymerization efficiency to avoid coking and carbonization caused by long-term heat treatment. By using ZnCl_2_ as the solvent and catalyst, the reaction time was greatly shortened from 40 h for ionothermal synthesis to 1 h for microwave-assisted synthesis [21]; however, this led to a very low degree of crystallinity of materials. X-ray diffraction (XRD) showed distorted layer structures and no obvious micropores. In order to improve the crystallinity of the synthesized polymer, trifluoromethanesulfonic acid (TfOH) was selected as Lewis acidic solvent and catalyst, because it has much stronger polarity than ZnCl_2_ and thus stronger microwave adsorption. Under the condition of air atmosphere at 11 °C, the polymerization time was further reduced to 30 min [22]. The hot-spot temperature was further increased by constant-power microwave irradiation, resulting in a much shorter reaction time of 30 s [10]. The degrees of conjugation and crystallization were greatly enhanced when using TfOH as solvent. However, the surface areas of such synthesized materials by TfOH were only 1–4 m^2^ g^−1^, which were much smaller than materials synthesized by ZnCl_2_ of ca. 1000 m^2^ g^−1^. As shown in Figure 4, the structure of CTF-1 synthesized by TfOH was staggered AB stacking, and the structure of CTF-1 formed with ZnCl_2_ was eclipsed AA stacking [31]. The staggered structure led to fewer interlayers through the micropores and a much smaller surface area. After significantly increasing the microwave power to 800 W, samples with relatively high surface areas of 300–700 m^2^ g^−1^ were observed. XRD confirmed that the structure of the samples synthesized with low microwave power was AA stacking rather than AB stacking [32].

Therefore, microwave-assisted synthesis can effectively reduce the reaction time and be conducted under an air atmosphere, which greatly improves the efficiency of catalyst preparation. More importantly, the reaction temperature is much lower than ionothermal synthesis, avoiding the carbonization of synthesized materials. CTF-1 synthesized by TfOH presented a high degree of crystallinity and conjugation, but the interlayer structure was staggered stacking with a tiny surface area, which was not conducive to the contact between the catalyst and reactants in photocatalytic reactions. Meanwhile, when decorating with cocatalysts on such materials with a low surface area, it was difficult to prevent the agglomeration of loading components, thus affecting the light adsorption of the catalyst [33]. While high-power microwave-assisted synthesis was able to observe catalysts with high surface area and easy to be increased for the piolet production, the control of carbonization was an issue [32]. Therefore, there is great potential for this method to be industrially utilized after more detailed optimizations and equipment design.

### 2.3. Dehydration Polycondensation 

**Figure 5:**
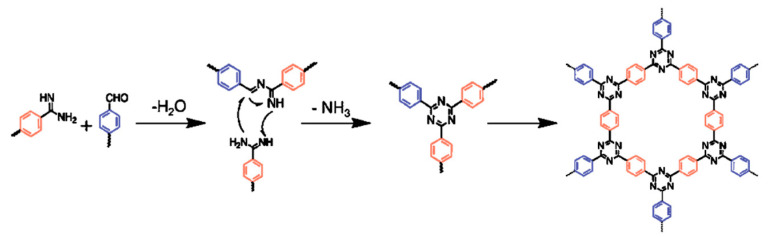
Polymerization of CTF-1 via a condensation reaction between an aldehyde and an amidine dihydrochloride over Cs_2_CO_3_ [23].

Dehydration of amidine [23] or amide [24] to form triazine require much lower activation energy than the trimerization of nitrile, thus reducing the polymerization temperature to 80–120 °C. The low reaction temperature is beneficial to prevent carbonization and, more importantly, functionalize the framework with thermolabile groups. A typical dehydration condensation route is shown in Figure 5 [23], using Cs_2_CO_3_ as catalyst and DMSO as solvent. A terephthalamidine molecule firstly reacted with an aromatic aldehyde to form a Schiff base. The reaction followed the nucleophilic addition reaction mechanism between primary amine and ketone, which was a basic chemical reaction widely utilized in the reaction to protect carbonyl groups. The Schiff base further reacted with another amidine molecule following Michael addition to form a triazine ring. A Michael addition reaction referred to a conjugated addition between an electrophilic conjugate electronic receptor and a nucleophilic electron donor. In the reaction, a formed Schiff base molecule was able to donate electrons to the conjugated double bond of the addition terephthalamidine molecule to form and remove -NH_3_ while generating a conjugated triazine motif via the rearrangement of electrons. The overall reaction time was around three days. The synthesized materials had an interlayer structure through holes and a relatively high surface area of ca. 700 m^2^ g^−1^. However, the crystallinity is relatively poor.

**Figure 6:**
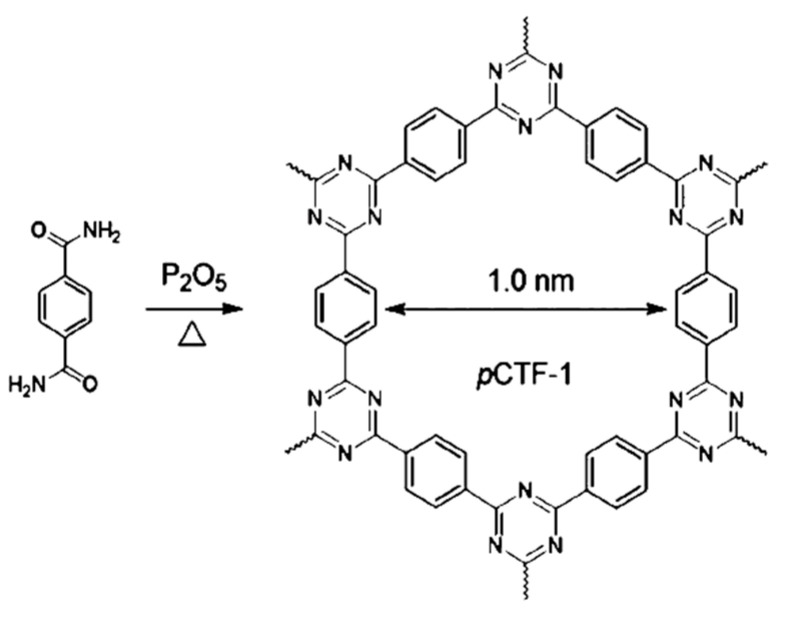
Dehydration polymerization of CTF-1 from the monomer terephthalamide over P_2_O_5_.

Terephthalamide is also utilized as a precursor for the synthesis of CTF-1. Under oxygen-free conditions and at 200 °C, P_2_O_5_ was able to dehydrate primary amide groups to form triazine motifs, as shown in Figure 6 [24]. The dehydration of amide was also a classic chemical reaction. In the reaction, electrons in the C=O double bonds were attracted by P atoms in P_2_O_5_ to form carbocations. Then, the lone-pair electrons of the nitrogen atoms were transferred to carbocations to remove hydrogen atoms and form C=N radicals. Triazine motifs were thus able to be generated via the trimerization of three C=N radicals. XRD results illustrated that the synthesized materials had a high degree of crystallinity and an eclipsed interlayer structure. The surface area was extremely high at 2000 m^2^ g^−1^, almost double compared to that of materials synthesized by ionothermal synthesis. Furthermore, the reaction temperature was only one-fourth of ionothermal synthesis; thus, carbonization hardly occurred during polymerization.

In conclusion, dehydration polycondensation effectively reduces the reaction temperature and avoids carbonization. Some of the synthesized materials have a high degree of crystallinity and large surface area, both of which are conducive to subsequent photocatalytic reactions.

### 2.4. Solution Polymerization 

**Figure 7:**
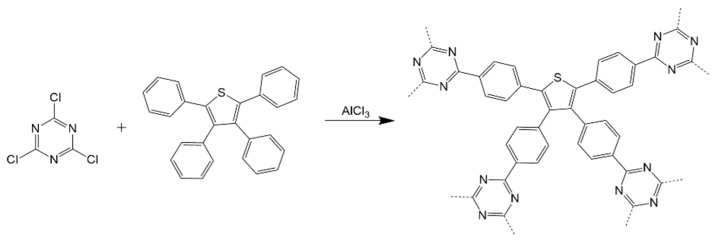
Polymerization of TTPT via Friedel–Crafts alkylation at 70 °C under reflux for 16 h [28].

Solution polymerization is another approach to synthesize CTFs under a relatively low reaction temperature of 0–150 °C via chemical reactions such as cyano trimerization [22], Friedel–Crafts alkylation [26], and Suzuki coupling [29]. The typical procedure [22] of cyano trimerization is to dissolve both aromatic nitrile and TfOH in CHCl_3_, respectively. Then, two solutions are mixed dropwise at 0 °C under a nitrogen-saturated atmosphere. After 8–12 h of reactions, CTFs are able to precipitate from the solution. However, their degrees of polymerization and crystallinity are at a very low level [25]. In addition, the obtained materials are also thermally unstable. The Friedel–Crafts alkylation reaction route is shown in Figure 7 [28]. Anhydrous AlCl_3_ was used as catalyst and CHCl_3_ served as solvent. Triazine precursors were able to electrophilically substitute the H atoms in aromatic rings at 70 °C to realize the coupling of triazine rings and aromatic rings. However, due to the poor selectivity of the above substantial reactions, triazine rings coupled with aromatic motifs at random sites. Therefore, the CTFs prepared by the Friedel–Crafts alkylation suffered from a low degree of crystallinity and conjugation, seriously distorted layers, and low surface areas [26,27,28].

**Figure 8:**
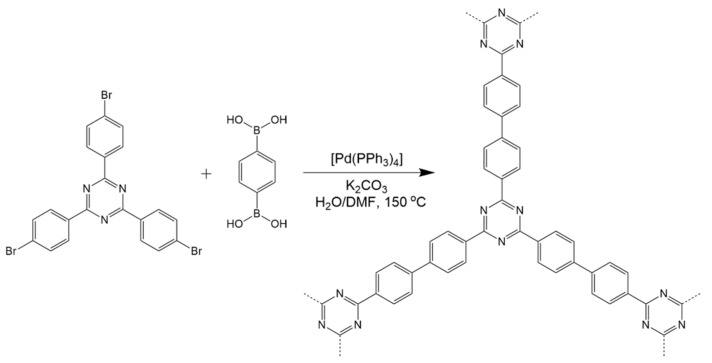
Polymerization of TTPT via Friedel–Crafts alkylation [29].

To control the selectivity of C-C coupling, Suzuki coupling is utilized in the synthesis of CTFs, as shown in Figure 8 [29]. Typically, brominated triphenyl triazine is able to cross-couple with boric acid or borate substituted aromatic motifs catalyzed by palladium complex under alkaline conditions at 150 °C to form CTFs. In the reaction, the catalyst Pd(0) complexes are oxidized by halogen atoms under strongly alkaline conditions to form electrophilic Pd(IV) complexes. Meanwhile, the organoboron complex reacts with a strong base to form nucleophilic borate. By the reductive elimination between the borate and Pd(IV) complex, C-C coupling is able to be achieved while regenerating Pd(IV) to Pd(0). The absence of annulation during Suzuki coupling enables the prepared sample to have a low level of defects in the molecular structure. Compared to samples synthesized by other methods with the same proposed molecular structure, CTFs prepared by Suzuki coupling had the largest bandgap, which was closest to the theoretical value predicted by the DFT calculation.

In summary, high crystallized CTFs are able to be constructed by C-C coupling by solution polymerization. On the one hand, C-C coupling is one of the classic reactions in catalyst synthesis. The reactions are conducted under relatively low temperatures, and there are sufficient studies on the side reactions. Therefore, the effective protection of functional groups is more possible than other polymerization paths, which provides a broader possibility for the optimization of the materials. On the other hand, during polymerization, the synthesis of triazine or aromatic rings is avoided, leading to fewer sp^3^ atomic defects in the ring structures than other synthesis methods. Thus, the synthesized materials are highly conjugated.

## 3. Optimization of CTFs for Photocatalytic Hydrogen Generation

**Figure 9:**
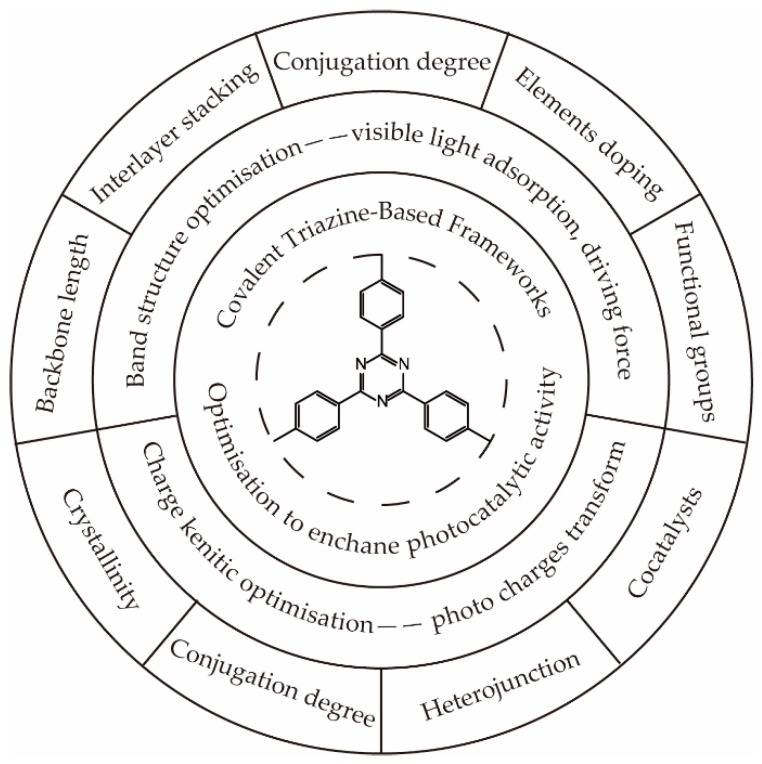
Functionalized optimization of the covalent triazine-based framework.

Although some efforts have been devoted to exploring efficient CTFs for solar-driven hydrogen generation, most of them focus on screening the block or loading cocatalysts to achieve high activity, ignoring the investigation of the mechanism. The application of highly conjugated polymer CTFs in photocatalysis was first predicted by first-principle calculations in 2015 [34]. According to the modeling results, CTFs had a suitable band structure that could effectively be excited by UV and visible light. The generated photoelectrons generated at −0.5 eV–−1 eV (vs. NHE) were sufficient to drive the proton reduction reaction to generate hydrogen. The energy of photogenerated holes was predicted between +2 eV and +1.2 eV (vs. NHE), which were able to oxidize water to form molecular oxygen. To improve the efficiency of photocatalytic hydrogen generation and investigate the relationship between the catalyst structure and performance, as presented in Figure 9, a great number of modification strategies have been performed to optimize the band structure [12,14,20,35,36,37,38,39,40] and charge dynamic process [10,15,41,42,43,44,45,46,47,48], thus broadening the visible light absorption, enhancing the driving force of hydrogen generation, and improving the transport and separation efficiency of photocharges.

### 3.1. Optimization of Band Structure to Broaden the Visible Light Absorption and Enhance the Driving Force for Hydrogen Generation

The band structure of photocatalysts determines the light response range and the energy of photogenerated charges. A smaller bandgap leads to a higher solar utilization rate. On the contrary, hydrogen generation is an endothermic reaction; thus, the energy of photogenerated charges must meet both the thermodynamic requirements and the overpotential for hydrogen generation. Therefore, the bandgap optimization of photocatalysts is necessary to strike a balance between light response and sufficient driving force.

#### 3.1.1. Optimize the Length of the Backbone

The distribution of aromatic and triazine motifs in CTFs determines the energy level of both the highest occupied molecular orbital (HOMO) and the lowest unoccupied molecular orbital (LUMO). Different quantities of benzene units are inserted between two triazine motifs to increase the length of the backbones. CTFs with different lengths of backbones were synthesized by dehydration polycondensation [23], Suzuki coupling [29] and nitrile trimerization [29]. The experimental results illustrated that the longer the backbone, the narrower the bandgap of CTFs, and the higher the efficiency of photonic utilization. More significantly, the number of aromatic spacers greatly impacted the energy of HOMO while maintaining the position of LUMO; thus, the optimized materials kept a similar driving force for hydrogen generation. However, with an increase in the aromatic proportion of the materials, the hydrophilicity deteriorated. Therefore, the mass transfer between the catalyst and water molecule became worse when increasing the length of the backbone by adding aromatic spacers. When two triazine units were spaced by two benzene motifs, the highest hydrogen generation rate was observed on the photocatalysts prepared by all three methods. The reported hydrogen generation rate was 7.4 µmol h^−1^ (>420 nm) for the catalyst prepared by polycondensation, 118.31 µmol h^−1^ (>420 nm) for the catalyst prepared by Suzuki coupling, and 132.15 µmol h^−1^ (>420 nm) for the catalyst prepared by nitrile trimerization. The activity difference among these samples was likely due to the different crystal structures and sp^3^ defects [7].

#### 3.1.2. Control the Interlayer Stacking

CTFs are 2D-layered conjugated materials. By controlling the stacking between layers, functional groups can have different interlayer interactions resulting in various distributions of electron clouds. CTF-0 with eclipsed AA stacking and staggered AB stacking were synthesized by ionothermal synthesis and microwave-assisted synthesis, respectively [12]. In the eclipsed stacked CTF-0, triazine corresponded to triazine, and benzene corresponded to triazine between two layers. Conversely, in the staggered stacked CTF-0, triazine corresponded to benzene between odd and even layers. Therefore, the interlayer n⟶π transition was possible in the staggered stacked CTFs. Both theoretical and experimental results indicated that interlayer excitation resulted in a more negative conduction band. Thus, the excited photoelectrons held higher energy to drive proton reduction. Therefore, the photocatalytic hydrogen evolution for the sample with AB stacking was 100 µmol h^−1^ (>420 nm), which was much higher than samples with AA stacking of 9 µmol h^−1^ (>420 nm) [12]. A redox exfoliation process was utilized to obtain ultrathin crystalline CTF. The overall thinness of r-CTF was only 1.2 nm, indicating 3–4 sheets in a piece of crystallized CTF, resulting in a photocatalytic hydrogen generation rate of 500 µmol h^−1^ (>420 nm) [49].

#### 3.1.3. Control the Degree of Polymerization

The degree of polymerization affects the number of terminal groups and the distance between layers. By controlling the reaction temperature, the ratio between ZnCl_2_ and monomer, and the polymerization time, the degree of polymerization was tuned via ionothermal synthesis [20]. The experimental results showed that the lower the degree of polymerization, the smaller the interlayer spacing, and the higher the hydrophilicity. However, the bandgap due to the π⟶π* transition widened, thus reducing light absorption efficiently. The benchmark photocatalytic activity of 10.8 (±2.8) µmol h^−1^ (solar simulator) was achieved via an almost linear oligomer PTO-300-15 with a molecular weight of 2000–3000 and an apparent quantum efficiency of 5.5 ± 1.1% (at 400 ± 20 nm).

#### 3.1.4. Element Doping

Heteroatom doping effectively changes the electron distribution of polymeric materials. In general, the doping of O [39], S [35], P [36], and other elements mainly contributed to HOMO electron distribution and changed the position of the valence band. Thus, it is possible to narrow the bandgap while maintaining the position of the conduction band to supply enough driving force for hydrogen generation. To dope sulfur into CTF-1, the polymerized CTF-1 was ground with sulfur powder and calcined at 250 °C for 1 h under a nitrogen atmosphere. The bandgap was sufficiently narrowed. Compared with the undoped sample, the photocatalytic activity for hydrogen generation was enhanced 4 times [35]. When replacing N atoms in CTF-1 with P atoms, a narrower bandgap was obtained as well, while the photocatalytic activity increased 5 times compared to the bare sample [36]. However, elemental doping may destroy the conjugated structure of the material and cause a great deviation in LUMO position as well. For the oxygen doping materials in particular, one study reported hydrogen had an evolution rate of only 0.5 µmol h^−1^ (>420 nm), which was much lower than the bare sample [39]. This could partially be explained by the halogen atoms doped samples. When treating CTF-1 with HCl, Cl atoms were successfully doped in the position between the C-N bond, showing much lower activity than the undoped sample [38]. On the contrary, when treated CTF-1 by NH_4_Cl, Cl atoms were only able to substitute H atoms in benzene motifs. The photocatalytic activity of the Cl-doped sample increased 7.1 times compared to the unmodified sample, and high quantum efficiency of 10.31% was achieved under 420 nm light irradiation [37]. By comparing the band structures of two Cl-doped samples, the conduction band slightly shifted to more negative as well when replacing H in benzene, although the overall bandgap was narrower than the unmodified sample, which thus gave more driving force for proton reduction while increasing the light absorption range. Fe^3+^ ions were also claimed to be doped into the CTF molecule structure. With the increase in Fe amount, the valence band position shifted more negatively, resulting in a 30 times improvement in the hydrogen generation rate than the bare sample of 30 µmol h^−1^ (>420 nm) [50]. Protonating CTFs by acid was a strategy to enhance hydrophilicity. The protonated CTF-1 presented a contact angle of 38.4°, which was only one-third of the bare sample, resulting in ca. 10 times enhancement of the hydrogen evolution rate [51].

#### 3.1.5. Functional Group Substitution

According to theoretical calculations, in a typical CTF molecule, HOMO is determined by the triazine motif, and LUMO is associated with the benzene unit. Therefore, proton reduction occurred on benzene sites [34]. However, due to the hydrogen bond between water and benzene, it is not conducive to dissociating water molecules. Therefore, the backbones of CTFs were functionalized to relocalize the electron distribution and optimize the active sites. Carbonyl carbazole, dibenzothiophene, and dibenzofuran were utilized as the aldehyde monomer for the dehydration polycondensation, thus introducing the above functional groups into the polymer backbone. The highest photocatalytic activity of 538 µmol h^−1^ (>420 nm) for hydrogen generation, and the quantum efficiency of 4.07% (at 420 nm) was over that of the carbazole-functionalized sample CTF-N [40]. The enhancement of the activity was due to the efficient charge transfer from the introduced functional groups to the triazine motifs and the narrowed bandgap. Furthermore, the active sites for proton reduction shifted from benzene to triazine. The lone-pair electrons in triazine were sufficient for the dissociation of water molecules, thus further enhancing the photocatalytic activity. Introducing two functional groups such as pyrazole and benzothiadiazole to change the activation sites for water oxidation and proton reduction, respectively, resulted in much higher hydrophilicity and better interaction between water and photocatalysts [12,13]. However, the backbone of the polymer became more distorted when introducing more functional groups, resulting in a low efficiency for electron transport. The hydrogen generation rate was relatively low at 50 µmol h^−1^ (>420 nm), and the quantum efficiency was 3.58% at 420 ± 20 nm. Thiophene and benzothiadiazole were introduced into the backbone of CTF via the cyano trimerization method. The HOMO level shifted greatly to narrower the bandgap while maintaining the LUMO level to enhance the light absorption. A hydrogen evolution rate of 112 µmol h^−1^ (>420 nm) was observed [52].

### 3.2. Control of Charge Dynamic to Enhance the Electron Transfer and Separation

A typical photocatalytic process can be described in three steps: (1) photon absorption by a semiconductor to generate excited charge carriers, (2) diffusion of photocharges to semiconductor surface, and (3) interfacial charge transfer between active sites, followed by the transfer to reactants. However, the excited photoelectrons and holes could instead recombine to determine the lifetime of the charge carriers. Furthermore, the lifetime of charge carriers directly impacted the diffusion length of photocharges. Therefore, the optimization of the charge dynamic by controlling the structure of photocatalysts was greatly significant.

#### 3.2.1. Enhance the Degree of Crystallinity

Both crystallinity and crystallite dimensions affect the efficiency of charge transfer and separation. Low crystallinity is due to the low stereoregularity and more defects in the molecule structure, both of which may result in the formation of recombination centers to obstruct charge transfer. On the contrary, high crystallinity lead to a large crystallite size. With an increase in crystallinity degree, the electrical conductivity raise, but the ionic conductance decrease. Thus, the diffusion of dissociated water species are limited [53]. Samples synthesized by triazine trimerization via solution polymerization had very poor crystallinity. The photocatalytic activity for hydrogen generation was very low, around 8 µmol h^−1^ (>420 nm) [25]. To mix the above sample with ZnCl_2_ and calcined under vacuum at 400 °C for 2.5 to 30 min, the crystallinity obviously increased. Among all treated samples, the material calcined 10 min presented the highest hydrogen generation rate of 26.8 µmol h^−1^ (>420 nm). More importantly, the quantum efficiency increased from 2.4% of the uncalcined sample to 6.4% of the treated sample. When calcining the sample for more than 10 min, the crystallinity further increased, while the photocatalytic activity decreased, probably attributed to the lower efficiency for the ionic conductance and the carbonization of the materials to block the light adsorption. By using a different base catalyst such as K_2_CO_3_, KOH, EtOK, and *^t^*BuOK, CTF-1s with different crystallinity were synthesized via Michael addition. The photocatalytic activity was positively correlated with the crystallinity of the synthesized CTF-1s [15]. The sample synthesized over *^t^*BuOK presented the highest hydrogen generation rate of 335 µmol h^−1^ (>420 nm) with a quantum efficiency of 7.4% at 420 nm. With the increase in the crystallinity, the lifetime of photocharges increased from 214.5 ps to 2630 ps, while the contact angles of water droplets on the surface decreased from 65.5° to ca. 5°. More importantly, when using Pt nanoparticles and NiP_x_ as the cocatalysts for proton reduction and water oxidation, respectively, efficient overall water splitting was observed with a hydrogen generation rate of 25.4 µmol h^−1^ [15]. A NaCl-KCl-ZnCl_2_ eutectic salt system reduced the polymerization temperature during ionothermal synthesis from 400 °C to 200 °C to avoid the carbonization process. Thus, a high crystallized sample was observed compared to that synthesized under high temperatures. A high degree of polymerization was also confirmed by FTIR. A hydrogen evolution rate of 60 µmol h^−1^ was achieved, which was 3 times higher than the sample with poor crystallinity synthesized by ZnCl_2_ only at 400 °C [54].

#### 3.2.2. Control the Degree of Conjugation

For CTFs that are layered polymers, the degree of conjugation represents the distortion of the polymeric material that originated from sp^3^ defects during synthesis. The higher the stereospecificity, the more efficient the interlayer charge transition and the within-layer charge transfer. The degree of conjugation of CTFs was able to be characterized by Raman spectra by calculating the ratio of sp^2^ to sp^3^. By controlling the microwave power, CTF-1s with different degrees of conjugation were synthesized by microwave-assisted synthesis. With the increasing conjugation degree, the efficiency of charge transfer greatly enhanced, resulting in a high hydrogen generation rate of 265 µmol h^−1^ with a quantum efficiency of 6.0% at 420 nm.

#### 3.2.3. Heterojunction Construction

A heterojunction is an interface region that results from the contact of two different semiconductors. Heterojunction construction allows photogenerated electrons and holes to be stored on different semiconductors, respectively, so that the hydrogen evolution and water oxidation reactions are able to be carried out on two counterparts, which promotes the transfer and utilization of photogenerated charges. CTF-1 was combined with CdS to form a type II heterojunction. According to electrochemical impedance spectroscopy and photoluminescence spectroscopy, the charge transfer of the composite was much stronger than that of two single materials, CdS and CTF-1. Using lactic acid as the sacrificial agent, the hydrogen evolution rate was 243 µmol h^−1^ (>420 nm), which was three times higher than that achieved by CdS nanoparticles [38]. Furthermore, as both conduction and valence band positions of CTF-1 were more positive than that of CdS, photogenerated electrons were directed to the conduction band of CdS, while photoholes transferred to the valence band of CTF-1, which effectively avoid the oxidative corrosion of CdS by photoholes. Therefore, the stability of the composite was much higher than CdS resulting in a 36 h continuous hydrogen generation without activity decay [41]. Similar phenomena were observed over the composite of MoS_2_ and CTF-1 with a quantum efficiency of ca. 7% at 420 nm [43].

**Figure 10:**
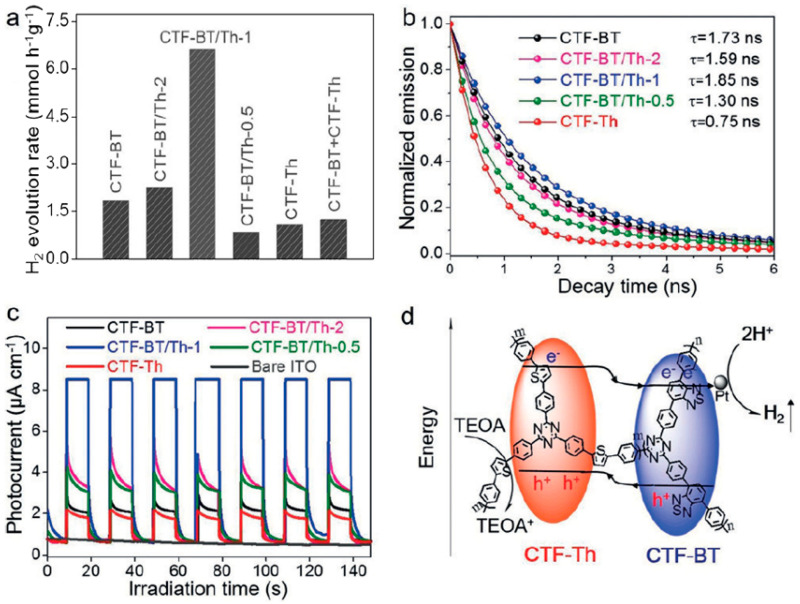
Photocatalytic activity for hydrogen evolution and efficiency of charge separation over a heterojunction photocatalyst of CTF-Th and CTF-BT [44]. (**a**) Photocatalytic activity for hydrogen evolution; (**b**) time-resolved photoluminance; (**c**) photocurrent responses and (**d**) illustration of the facilitated charge-carrier separation across the covalently interconnected molecular heterostructure.

Thiophene and benzothiadiazole were introduced at various positions of the backbones of CTFs. Thus, a nanocomposite was formed as a type II heterojunction, as shown in Figure 10. Photoelectrons were concentrated in the benzothiadiazole side as the different HOMO and LUMO structures of two parts of the backbones. Therefore, the density of photoelectrons was enhanced. Meanwhile, as the polymer was based on benzene and triazine motifs, the charge transfer efficiency between the two parts was much higher than the charge transfer in conjugated polymers and metal-based compounds such as CdS, MoS_2_, etc. Therefore, such an amazing structure allowed it to present a hydrogen generation rate of 330 µmol h^−1^ (>420 nm) and a quantum efficiency of 7.3% at 420 nm [44]. When using N-ethylcarbazole to replace thiophene in the nanocomposite, the energy band difference between electron donor and acceptor was increased. Therefore, the hydrogen generation rate was further enhanced to 966 µmol h^−1^ (>420 nm), and the quantum efficiency was 22.8% [45].

#### 3.2.4. Cocatalyst Deposition

Noble metals are typically considered cocatalysts for hydrogen generation. Among them, the free Gibbs energy for hydrogen dissociation is close to 0 for Pt, Pd, and Rh, which is much smaller than that on the surface of Au, Ag, Cu, and Ni. Therefore, Pt, Pd, and Rh are loaded. Meanwhile, the work functions of such noble metals are between the conduction and valence bands of CTFs. Therefore, a Schottky junction is able to be formed to direct photoelectrons to the cocatalyst. The majority of cocatalyst utilized in the previously reviewed measurements were Pt nanoparticles. There were mainly three deposition paths, and all start from chloroplatinic acid: (1) impregnation of chloroplatinic acid with CTFs and calcinated in the air to decomposed; (2) utilizing sodium borohydride to reduce platinum in solution; (3) photodeposited platinum in situ by photogenerated electrons. All three routes were able to anchorite Pt nanoparticles on the surface of CTFs. However, due to the high conjugated surface of CTFs, it was difficult to form chemical bonds between Pt nanoparticles and CTFs. Thus, the efficiency of electron transfer was limited. In order to enhance the metal–semiconductor contact and improve the electron transfer, black phosphorus served as a bridge to connect the Pt nanoparticles and CTFs by forming N-P-Pt chemical bonds. The hydrogen generation rate over Pt/P/CTF-1 was 5 times higher than that of Pt/CTF-1 [46]. However, black phosphorus had a band structure, thus forming a type I heterojunction when deposited on CTF-1. Therefore, both photogenerated electrons and holes would transfer to black phosphorus, resulting in the recombination [47,55]. To overcome the drawbacks of the band structure of black phosphorus, one of the carbon atoms in the benzene motif was replaced by a nitrogen atom to form a bipyridine-like structure with triazine. The bidentate ligands were able to interact with Pt and Pd chlorides from the chemical bond between CTFs and noble metal active centers. The hydrogen generation rate of Pd decorated nitrogen-doped CTF reached 106 µmol h^−1^ (>420 nm) [48]. Another strategy was to directly utilize Ni_2_P as the cocatalyst to get a better interaction for charge transfer. However, as the higher driving force for the hydrogen evolution over Ni sites the hydrogen evolution over Ni_2_P/CTF was similar to Pt/CTF [56]. rGO was also investigated as the cocatalyst to enhance the charge separation, the hydrogen evolution was of 20–30 µmol h^−1^ (>420 nm), which was more than 10 times higher than the bare sample under the identical conditions [57,58].

## 4. Conclusions

As a novel polymeric photocatalyst, CTFs have been widely applied in photocatalytic hydrogen generation due to their suitable band structure, highly conjugated structure, and strong stability. This manuscript reviewed the development of CTFs applied for hydrogen generation, followed by the presentation of efficient optimization strategies for structural engineering. In order to improve the hydrogen generation rate, the crystallinity, degree of conjugation, interlayer stacking, and band structure were optimized by controlling the reaction processes, introducing functional groups and constructing heterojunctions. However, the understanding of CTFs for photocatalytic hydrogen generation is still in a relatively infant stage, especially for the control strategies of hydrophilicity, band structure, and charge dynamics. Overall, the investigation of CTFs on photocatalytic hydrogen evolution could be further improved as follows: (1) design and synthesis of new monomers for the polymerization of CTFs to control the physical and chemical properties of CTFs at the molecular level to enhance the capability of water adsorption, reduce the activation energy of proton reduction, increase the light absorption range, increase the driving force, and extend the lifetime of photocharges; (2) design and synthesis of new CTFs with two kinds of functionalized backbones in one framework to form a highly conjugated heterojunction composite to enhance charge separation and transfer; (3) construct a Z-scheme structure with electron shuttles and other semiconductors to enhance the driving force; (4) optimize the surface of CTFs to improve the connection with noble metal cocatalysts; (5) utilize advanced characterization (e.g., X-ray absorption spectroscopy, transient absorption spectroscopy) and first-principle calculations to investigate the reaction mechanism; (6) decorate proper cocatalysts on optimized CTF-1 to enhance the photocatalytic efficiency of the overall water splitting.

## Data Availability

Not applicable.
